# Comparison of the mechanical properties of different skin sites for auricular and nasal reconstruction

**DOI:** 10.1186/s40463-017-0210-6

**Published:** 2017-04-18

**Authors:** M. F. Griffin, B. C. Leung, Y. Premakumar, M. Szarko, P. E. Butler

**Affiliations:** 1 Division of Surgery & Interventional Science, University College London (UCL), London, UK; 2 Anatomy Department, St Georges University, London, UK; 3 Plastic & Reconstructive Surgery Department, Royal Free Hospital, London, UK

**Keywords:** Biomechanics skin, Skin flap, Nasal, Auricular, Young elastic modulus

## Abstract

**Background:**

Autologous and synthetic nasal and auricular frameworks require skin coverage. The surgeon’s decides on the appropriate skin coverage for reconstruction based on colour matching, subcutaneous tissue thickness, expertise and experience. One of the major complications of placing subcutaneous implants is the risk of extrusion (migration through the skin) and infection. However, knowledge of lessening the differential between the soft tissue and the framework can have important implications for extrusion. This study compared the mechanical properties of the skin commonly used as skin sites for the coverage in auricular and nasal reconstruction.

**Methods:**

Using ten fresh human cadavers, the tensile Young’s Modulus of the skin from the forehead, forearm, temporoparietal, post-auricular and submandibular neck was assessed. The relaxation rate and absolute relaxation level was also assessed after 90 min of relaxation.

**Results:**

The submandibular skin showed the greatest Young’s elastic modulus in tension of all regions (1.28 MPa ±0.06) and forearm showed the lowest (1.03 MPa ±0.06). The forehead demonstrated greater relaxation rates among the different skin regions (7.8 MPa^−07^ ± 0.1). The forearm showed the lowest rate of relaxation (4.74 MPa^−07^ ± 0.1). The forearm (0.04 MPa ±0.004) and submandibular neck skin (0.04 MPa ±0.005) showed similar absolute levels of relaxation, which were significantly greater than the other skin regions (*p* < 0.05).

**Conclusions:**

This study provides an understanding into the biomechanical properties of the skin of different sites allowing surgeons to consider this parameter when trying to identify the optimal skin coverage in nasal and auricular reconstruction.

**Electronic supplementary material:**

The online version of this article (doi:10.1186/s40463-017-0210-6) contains supplementary material, which is available to authorized users.

## Background

Craniofacial auricular and nasal cartilage defects including those caused by cancer, trauma, burns or congenital deformities are restored by transferring autologous cartilage from elsewhere in the body and shaping the tissue for the specific anatomical defect [1]. However, due to limitations in availability of suitable tissue, resorption of the tissue with time, surgeons have utilised synthetic materials to replace the cartilage defect [1]. Several materials have been investigated to replace the cartilaginous framework including silicone, porous polyethylene (Medpor ®) and polyfluorethylene (Gore-tex) with different mechanical properties [1]. However, research is still on going to develop materials to replace the cartilaginous frameworks due to the high levels of infection and extrusion with currently available materials [1]. Whether surgeons use autologous or allografts, the cartilaginous framework requires soft tissue coverage. Surgeons choose their skin coverage based on colour match, tissue availability, pedicle quality and personal experience and expertise [2]. Currently, used skin flaps to cover an auricular implants include the temporoparietal and postauricular mastoid due to the good colour match being anatomically close to the ear [3, 4]. For nasal reconstruction the forehead is the gold standard due to the close colour match and subcutaneous thickness. The forearm skin flap can also be used if the forehead flap is unavailable [5, 6].

Implant extrusion is a devastating complication of using allografts, where the implant migrates through the subcutaneous skin meaning the patient requires further surgical intervention [7]. The cause of extrusion of implants is unpredictable and not well understood. The mechanism of implant extrusion has been suggested to result from infection, patient co-morbidities and implant characteristics [7, 8].

One technique implemented by craniofacial surgeons when utilising synthetic materials for auricular or nasal reconstruction is stress shielding. This involves wrapping the implant with a layer of autologous tissue to reduce the contact stress subjected to the overlying skin by the implant. Several reports have shown that fascia covered frameworks reduce the stress of the implant on the surrounding skin tissue and aid in the prevention of extrusion [8, 9].

Similarly, it has been shown that if an implant is manufactured with similar mechanical properties to the surrounding tissues, then shear stress can be avoided, preventing micro-movement, risk of infection and extrusion of the implant [10–12]. Therefore, for researchers developing new auricular and nasal implants, it would be important to consider the mechanical properties of the skin coverage that is going to be utilised by the surgeon to cover the implant [10, 11]. However, the mechanical property of the skin of different sites for auricular and nasal reconstruction has not been investigated to date. We have created a reliable and simple protocol to analyse the tensile mechanical properties of human skin tissue [13, 14]. Tensile testing is a standard method of testing mechanical properties of skin tissue [13, 14]. The aim of this study was to analyse the mechanical and histological properties of common skin flaps for coverage in auricular and nasal reconstruction including the forehead, forearm, temporoparietal, post-auricular and submandibular neck.

## Methods

### Skin sample preparation

A total of ten fresh frozen cadaveric human heads were obtained for skin sampling and full ethnical approval was obtained. The samples included ten males, average age of 84 (range 77 to 94 years), average weight of 102 pounds (range 88 to 130 pounds), Caucasians without with any underlying skin condition or significant comorbidities.

Four regions were selected for skin sampling in each cadaver head: forehead, temporoparietal, post-auricular mastoid and submandibular neck. A total of fourty skin samples were obtained from the ten heads (1 samples per site). Forearms were also taken from the same cadavers. The position and orientation of the skin samples were kept constant, and were designed longitudinally parallel to Langer’s lines. Skin samples were 10 mm × 50 mm and accurately measured with digital vernier calipers. The subcutaneous fat is removed from the skin sample, leaving only the epidermis and dermis. A digital vernier caliper was used to measure the thickness in the different regions prior to mechanical testing.

### Skin extraction protocol

The skin samples were marked out on the cadaveric heads using a repeatable method as follows (Additional file 1: Figure S1). The forehead skin was taken 20 mm from the superior nasal bridge indentation and spanned 50 mm across the forehead with a 10 mm height, following the forehead lines. The temporoparietal skin was measured 10 mm above the zygomatic arch and originated 20 mm posterior to the lateral orbital rim, then extended 50 mm posteriorly towards the superior border of the ear with a 10 mm height. The post-auricular mastoid skin was taken from 10 mm posterior to the auricular ear attachment and originated at corner of the ear lobe, then extended towards to the back of head, 50 mm in length and 10 mm in width. The submandibular neck skin was measured 10 mm below and parallel to the submandibular border, extending 50 mm in length and 10 mm in height. The forearm skin was split into anterior, middle and posterior skin. A square paddle was drawn 15 cm posterior from the ulnar styloid and divided into three equal sections (Additional file 1: Figure S1). Anterior forearm was considered the section nearest the wrist joint and posterior was the nearest the elbow joint. Each section was then divided 50 mm in length and 10 mm width per section (*n* = 5 per section of forearm).

Skin samples were immediately stored into 20 ml of Phosphate Buffer solution (PBS) contained in 100 ml bottles after initial cutting and fat stripping, followed by storage in the freezer at −80 °C. The storing process was conducted simultaneously for all the samples, which avoided any shrinkage of the skin. The thawing process prior to the biomechanical testing was carried out by immersing the skin samples in a water bath set at 37.5–38 °C that was checked manually with a digital thermometer, for 15 min. The skin samples were then dried with clean paper towels and were checked for achievement of room temperature, 25–26 °C. Once the skin samples reached room temperature, the samples were then ready for mechanical testing.

### Mechanical testing protocol

#### Biomechanical testing

Skin samples were measured in uniaxial tension using a Mach-1 materials testing machine (Biomomentum, Canada) as described by our institution, Wood et al., 2012 [13]. Each skin sample was orientated and immobilised between two clamps, one fixed to a 10 kg load cell and the other to the immovable base plate. No slippage was observed as the samples were fixed in a commercial jig suing ‘finger tight’ tightness. The resulting area to be tested is then stable of 1 cm by 4 cm. The sample was loaded to 3000 g at 1 mm/s. After the 29.42 N load was reached, the tissue was allowed to relax for 1.5 h (a time point sufficient for control skin to reach equilibrium). Calculation of the Young’s modulus in tension was then performed as described previously [13] and demonstrated in Additional file 2: Figure S2. The rate of skin relaxation was evaluated by measuring the final 200 s of the experiment (stress (MPa) over time (seconds)) (Additional file 2: Figure S2). The final stress relaxation was calculated by measuring the final skin stress after 90 min (Additional file 2: Figure S2). During experimentation petroleum jelly was applied to prevent desiccation of the skin samples.

#### Histological analysis

A 1 × 1 cm^2^ samples was taken from the donor before testing. Tissue was formalin-fixed, paraffin-embedded and sectioned at 8 μm. Each section was analysed using H&E, Masson’s Trichrome, and Miller’s elastin stains were conducted according to standard protocols, then photographed with a Zeiss Axioplan microscope.

#### Statistical analysis

Inter-treatment comparisons were analysed statistically using one-way analysis of variance (ANOVA) with Tukey HSD post-hoc analysis (JMP, v10; North Carolina, USA). The average and standard deviation (SD) was calculated. Significance was described as *p* < 0.05. Kaleidagraph (v.4.1, Pennsylvania, USA) was used for graphically representing data.

## Results

### Biomechanical testing

**Fig. 1:**
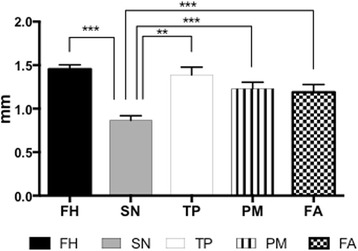
Average thickness of the different skin sites prior to mechanical testing. Key; FH; Forehead, SN; Submandibular Neck, TP; Temporoparietal Neck, PM; Postauricular Mastoid, FA; Forearm (* *p* < 0.05, ** *p* < 0.01, *** *p* < 0.001)

Prior to mechanical analysis, the average thickness of the different skin sites was measured using electronic callipers. Figure 1 shows the forehead (1.4 mm ±0.05 SD) and temporoparietal region (1.39 mm ±0.09 SD) were the thickest of the skin regions. The submandibular neck was the significantly thinnest of all the skin regions (0.87 mm ±0.05 SD). The postauricular mastoid and the forearm region showed similar thickness (PM 1.23 mm ±0.07 SD and FA 1.19 mm ±0.09 SD).

**Fig. 2:**
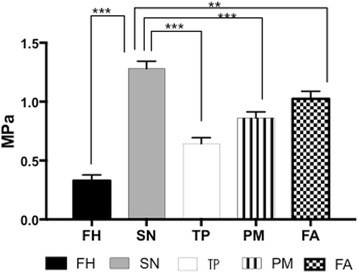
Young’s elastic modulus of the different skin sites under tension. Key; FH; Forehead, SN; Submandibular Neck, TP; Temporoparietal Neck, PM; Postauricular Mastoid, FA; Forearm (* *p* < 0.05, ** *p* < 0.01, *** *p* < 0.001)

The tensile Young’s elastic modulus was varied among the different skin sites (Fig. 2). The submandibular skin showed the greatest Young’s elastic modulus of all the regions (1.28 MPa ±0.06 SD, *p* <0.01). The forearm had a higher Young’s modulus than all regions except for the submandibular skin (1.03 MPa ±0.06 SD). The postauricular mastoid had a higher Young’s modulus than the forearm (0.86 MPa ±0.05 SD and the temporoparietal skin (0.65 MPa ±0.05 SD). The forehead had the lowest Young’s modulus in tension of all the regions (0.33 MPa ±0.04 SD). The Young’s elastic modulus of the forearm of the anterior, middle and posterior sections was also evaluated. The anterior forearm had a greater Young’s elastic modulus than the posterior forearm in tension but showed no significant difference (Additional file 3: Figure S3).

**Fig. 3:**
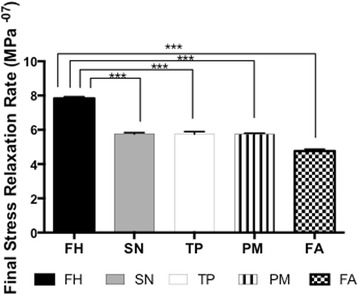
Final Stress Relaxation Rate of the different skin sites. Key; FH; Forehead, SN; Submandibular Neck, TP; Temporoparietal Neck, PM; Postauricular Mastoid, FA; Forearm (* *p* < 0.05, ** *p* < 0.01, *** *p* < 0.001)

The forehead showed a significantly greater rate of relaxation among the different skin regions (Fig. 3) (7.8 MPa^−07^ ± 0.1 SD, *p* <0.001). The submandibular neck (5.75 MPa^−07^ ± 0.08 SD), temporoparietal (5.7 MPa^−07^ ± 0.15 SD) and postauricular mastoid (5.7 MPa^−07^ ± 0.07 SD) skin showed similar rates of relaxation. The forearm showed the lowest rate of relaxation among the different skin regions (4.74 MPa^−07^ ± 0.1 SD).

**Fig. 4:**
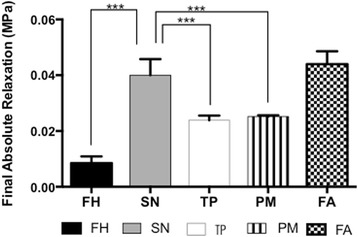
Final Absolute Relaxation of the different skin sites. Key; FH; Forehead, SN; Submandibular Neck, TP; Temporoparietal Neck, PM; Postauricular Mastoid, FA; Forearm (* *p* < 0.05, ** *p* < 0.01, *** *p* < 0.001)

The forearm (0.04 MPa ±0.004 SD) and submandibular neck skin (0.04 MPa ±0.005 SD) showed similar absolute levels of relaxation, which were significantly greater than the other regions of the skin (*p* <0.001) (Fig. 4). The temporoparietal (0.02 MPa ±0.001 SD) and postauricular skin (0.02 MPa ±0.0004 SD), showed similar levels of relaxation. The forehead showed the lowest absolute relaxation level among the different skin regions (0.008 MPa ±0.002 SD).

### Histological analysis

The H&E stain was used to subjectively assess the architecture of the skin from the different sites (Additional file 4: Figure S4). All samples showed a similar morphological architecture. The epidermis was a similar size among the skin sites. The papillary dermis and the deeper reticular dermis were also similar. There was also a similar quantity of vasculature in the dermis among the different sites. The submandibular neck showed muscle fibres of platysma, which was not present in the other skin sites.

Massons Trichome was used to subjectively assess the collagen staining in the different skin sites (Additional file 4: Figure S4). Collagen is the predominant constituent in the dermis accounting for 77% of its dry weight [15]. The collagen was stained as green and muscle as red. On subjective assessment the submandibular, post-auricular mastoid and forearm skin showed higher collagen staining than the forehead and temporoparietal regions. The submandibular skin showed think dense collagen bundles in the dermal regions.

Millers Elastin stain was used to subjectively assess the elastin and collagen content in the skin tissue regions (Additional file 4: Figure S4). Elastin fibres were stained black and collagen red. Elastin is responsible for the elasticity and the recoiling of the skin [15]. The elastin in the skin showed a similar pattern to the Massons Trichome where the collagen staining was greater in the submandibular skin, post-auricular mastoid and forearm than the forehead and temporoparietal regions. The elastin staining was also lower in the temporoparietal region than the other skin regions. The forehead showed the greatest level of elastin staining.

## Discussion

The aim of this study was to understand the mechanical properties of skin of common sites used for auricular and nasal framework coverage to help surgeons choose skin coverage appropriately to avoid extrusion and infection complications.

The submandibular neck skin demonstrated the highest Young’s elastic modulus in tension followed by the forearm skin (Fig. 2). The temporoparietal and post auricular skin showed a similar Young’s elastic modulus, which was less than the submandibular and forearm. The forehead skin showed the lowest Young’s elastic modulus in tension. Skin is known as an elastic tissue, deforming in response to applied forces [15, 16]. Histology using H&E showed no significant changes in the architecture that may account for these changes.

Collagen fibres are arranged with their long axis parallel to the relax skin tension lines, which are visible on the surface of the skin as the main lines [17]. Collagen fibres within the dermis are responsible for the tensile strength of skin. The submandibular neck, forearm and the temporoparietal skin showed greater staining for collagen using Massons Trichome stain, which may account for the changes in the observed tensile mechanical behaviour.

The relaxation rate was the greatest in the forehead skin (Fig. 3). The submandibular, temporoparietal and post-auricular skin showed similar rates of relaxation. The forearm skin demonstrated the slowest relaxation rate. The absolute relaxation rate was the greatest in the forehead, with similar values among the temporoparietal and neck skin (Fig. 4). The forearm skin demonstrated the lowest absolute relaxation rate (Fig. 4). Elastin is another structural constituent of the skin dermis. Elastin allows for skin’s ability to return to its original shape after loading [18]. The forearm showed the greatest elastin content of all the skin regions by Millers Elastin stain, which may account for this region showing the greatest relaxation rate and absolute relaxation level (Additional file 4: Figure S4).

When comparing the biomechanical values for the skin in tension to the literature, there is variation among authors, which is to be expected with biological tissues. The variability may be due to the biological tissue’s test conditions and variability between subjects. Ni Annaidh et al. compared the results in the literature to date of excised human skin, reporting the Youngs elastic modulus to range from 2.9 to 150 MPa irrespective of different donor sites [19]. The authors observed that the tensile properties of skin from the human back showed an elastic modulus of 83.3 ± 34.9 MPa [19]. Jacquemond et al. compared the in vitro tension of forehead and arm, observing an elastic modulus of 19.5–87.1 MPa [20]. Other than the reasons already discussed to account for the differences in values in different studies for the biomechanics of skin, it may be due to the properties of the skin changing once removed from the body with different storage and preparation protocols.

They are multiple different nasal and auricular implants available with different mechanical properties [1]. The relevance of our study to the clinical setting is providing knowledge of the mechanical and structural properties of different skin sites to understand which may be more compatible for certain nasal or auricular implants. This study may indicate that submandibular skin would be a suitable choice for reconstruction with a stiff implant as higher stress is likely to be required to cause skin breakdown and consequential implant extrusion. Forearm skin also showed a high Young’s modulus and could also be suitable for stiff implants. Forehead skin showed the lowest Young’s elastic modulus, demonstrating stiff implants may cause strain on this skin coverage. Forehead skin also showed the greatest rates of relaxation among the regions, which may mean the forehead skin may be able to relax when stressed over implants to a greater degree than other skin sites. Forehead skin also showed the lowest absolute final stress, which could indicate the implants would be under less stress from the skin with this choice of coverage. We have provided a foundation into the understanding of skin site’s mechanical properties; however further knowledge of implant mechanics and the interaction with the overlying skin would be required to influence future clinical care. Thus in the future, the mechanical tensile range of the skin sites could be considered along with colour match, hair presence, available skin volume required to better prevent extrusion of nasal and auricular replacements. Our future work will be to further understand how to pair implants with the mechanical properties of the overlying skin coverage.

Due to limitations in sample numbers, future work will be to take into consideration of different cofounding factors that may affect the mechanical properties of the skin including age, comorbidities and different skin types. The preparation of the skin regions included the removal of subcutaneous fat to prevent discrepancies within the data set. Future work will endeavour to account for this layer of skin, as this would be present in skin flaps in the clinical setting and taken into consideration for flap pliability and robustness of the random dermal plexus blood supply. All skin samples underwent two freeze-thaw cycles, which may have affected the mechanical properties. However, compared to other skin preservation methods, such as formaldehyde or embalming, freezing induces the least structural and mechanical changes to the skin [21]. Furthermore, skin has shown to be anisotropic, with Langer demonstrating that in the 1861, skin has natural lines of tension [19]. In this study, to avoid sample bias skin was oriented parallel to Langer lines. In future studies, it will be important to increase the sample size to allow for testing in different directions.

## Conclusions

In conclusion, we have demonstrated that human skin from different skin sites for auricular and nasal framework skin coverage behaves differently under tension loads. From this mechanical study, submandibular and forearm skin would be able to cover stiff implants well and forehead skin would cover soft implants well. Surgeons should consider the mechanical behaviour of the skin site in addition to donor site morbidity, colour and surgeon preference when choosing appropriate skin coverage in auricular and nasal reconstruction.

## Additional files

Additional file 1: Figure S1. Schematic diagram to illustrate how the skin samples were excised during the study. [A] Forehead excision. [B] Submandibular Neck, Temporoparietal Neck, and Postauricular Mastoid exicision. [C] Forearm excision. (TIFF 1521 kb)

Additional file 2: Figure S2. Loading data of a representative skin samples. [A] Analysis of initial load resistance data allowed the evaluation of the Young’s elastic modulus. [B] Measuring the rate of stress relaxation over the last 200 s allowed the determination of the final rate of relaxation, and measuring the stress level at the end of the 90 min relaxation period allowed the calculation of the final absolute relaxation (last point on B). (TIFF 1521 kb)

Additional file 3: Figure S3﻿. Tensile Young’s elastic modulus of the different forearm sites including the anterior, middle and posterior sites. (TIFF 1521 kb)

Additional file 4: Figure S4. Histological analysis of the different skin sites by H&E, Massons Trichome and Millers Elastin staining. [A] H&E [B]. Massons Trichome [C]. Millers and Elastin. Key; FH; Forehead, SN; Submandibular Neck, TP; Temporoparietal Neck, PM; Postauricular Mastoid, FA; Forearm. Scale bar = 50 μm. (ZIP 2133 kb)
