# Supplementary figures and images for: Comparison of the mechanical properties of different skin sites for auricular and nasal reconstruction
Source: J Otolaryngol Head Neck Surg. 2017 Apr 18;46:33. doi: 10.1186/s40463-017-0210-6 (PMC5395887; doi:10.1186/s40463-017-0210-6)

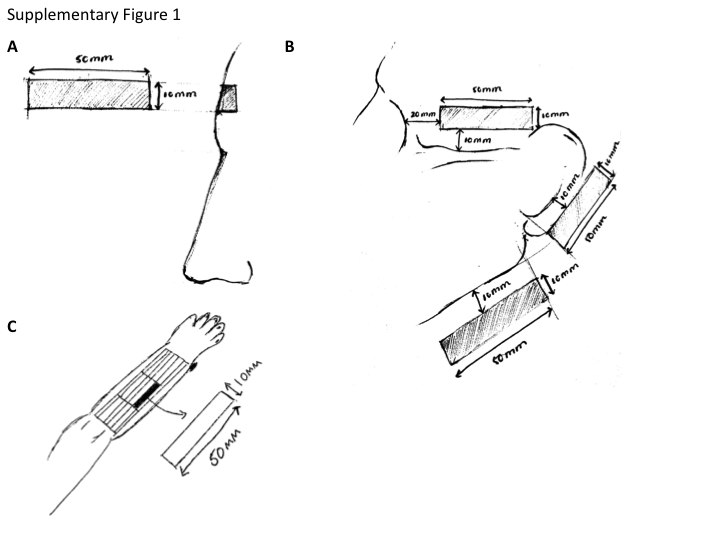

Supplement: Supplementary file 1 — Schematic diagram to illustrate how the skin samples were excised during the study. [A] Forehead excision. [B] Submandibular Neck, Temporoparietal Neck, and Postauricular Mastoid exicision. [C] Forearm excision. (TIFF 1521 kb) [file 40463_2017_210_MOESM1_ESM.tiff]

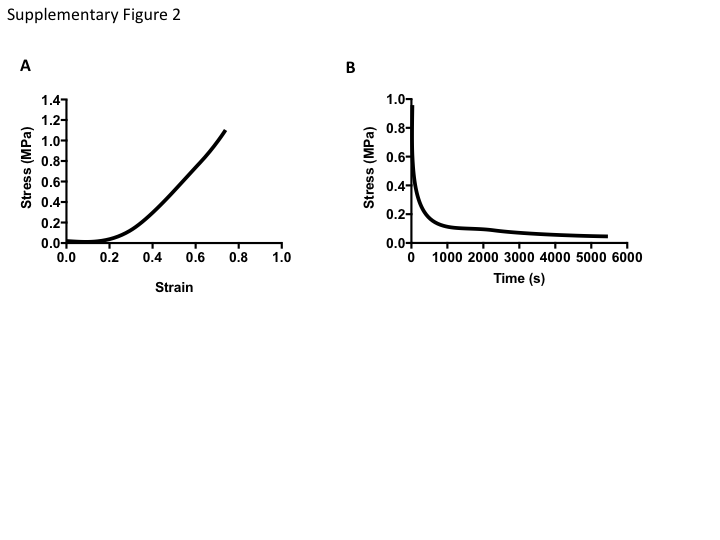

Supplement: Supplementary file 2 — Loading data of a representative skin samples. [A] Analysis of initial load resistance data allowed the evaluation of the Young’s elastic modulus. [B] Measuring the rate of stress relaxation over the last 200 s allowed the determination of the final rate of relaxation, and measuring the stress level at the end of the 90 min relaxation period allowed the calculation of the final absolute relaxation (last point on B). (TIFF 1521 kb) [file 40463_2017_210_MOESM2_ESM.tiff]

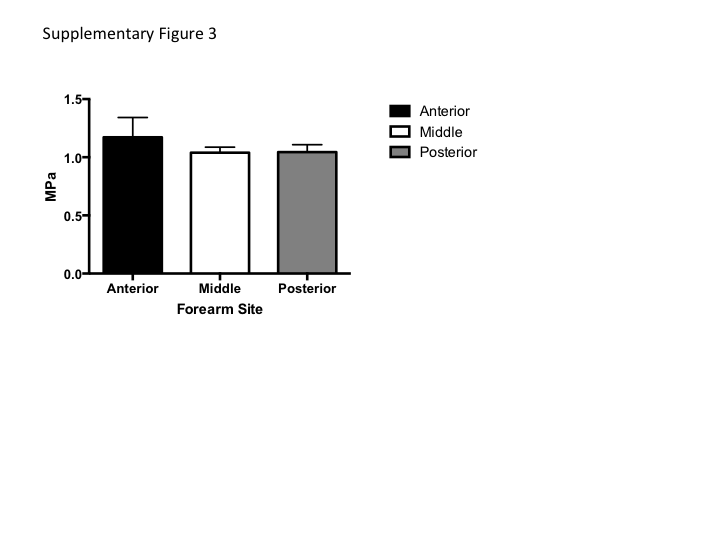

Supplement: Supplementary file 3 — Tensile Young’s elastic modulus of the different forearm sites including the anterior, middle and posterior sites. (TIFF 1521 kb) [file 40463_2017_210_MOESM3_ESM.tiff]

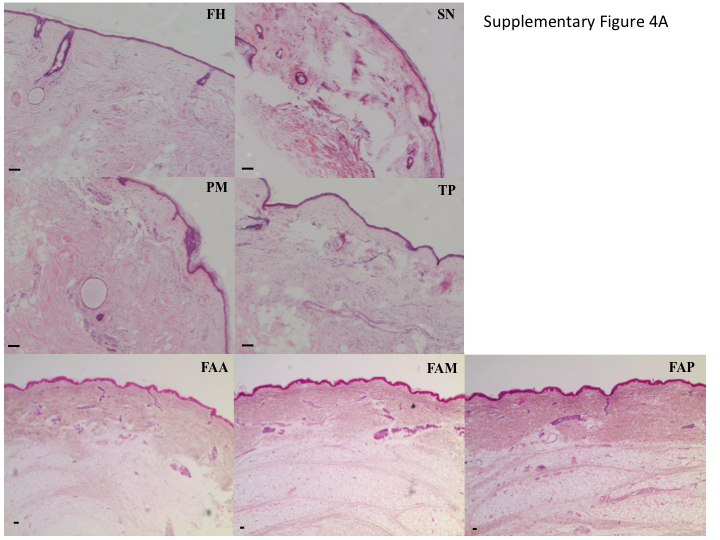

Supplement: Supplementary file 4 — Histological analysis of the different skin sites by H&E, Massons Trichome and Millers Elastin staining. [A] H&E [B]. Massons Trichome [C]. Millers and Elastin. Key; FH; Forehead, SN; Submandibular Neck, TP; Temporoparietal Neck, PM; Postauricular Mastoid, FA; Forearm. Scale bar = 50 μm. (ZIP 2133 kb) [file 40463_2017_210_MOESM4_ESM.zip › Slide08.tiff]

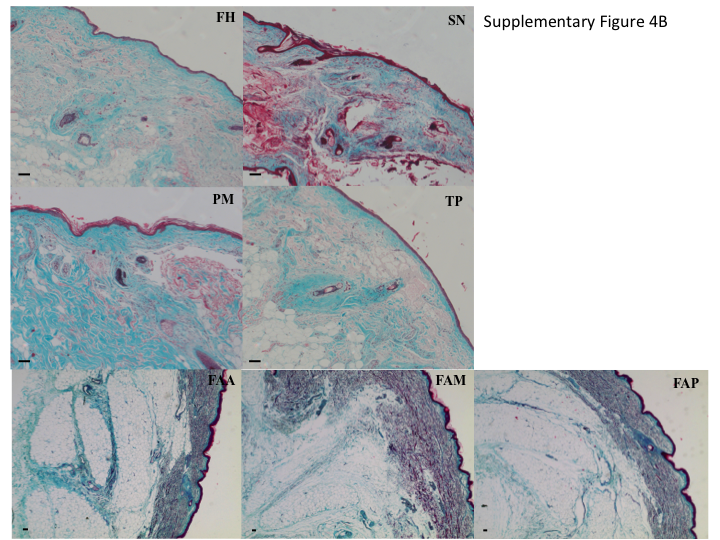

Supplement: Supplementary file 4 — Histological analysis of the different skin sites by H&E, Massons Trichome and Millers Elastin staining. [A] H&E [B]. Massons Trichome [C]. Millers and Elastin. Key; FH; Forehead, SN; Submandibular Neck, TP; Temporoparietal Neck, PM; Postauricular Mastoid, FA; Forearm. Scale bar = 50 μm. (ZIP 2133 kb) [file 40463_2017_210_MOESM4_ESM.zip › Slide09.tiff]

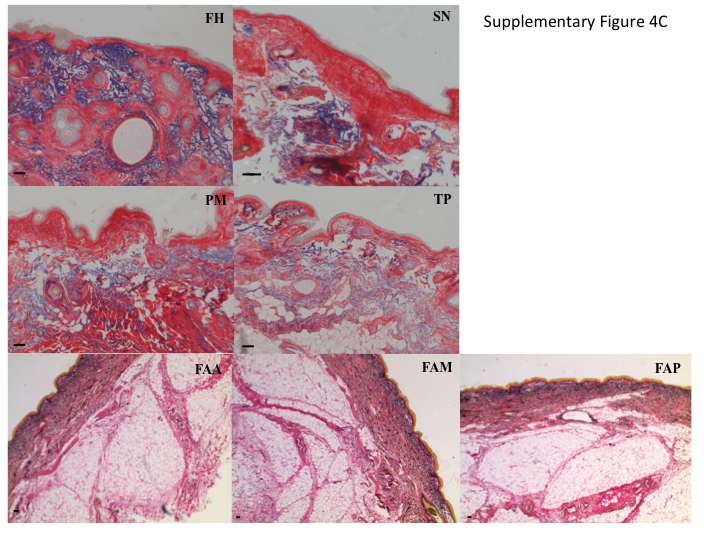

Supplement: Supplementary file 4 — Histological analysis of the different skin sites by H&E, Massons Trichome and Millers Elastin staining. [A] H&E [B]. Massons Trichome [C]. Millers and Elastin. Key; FH; Forehead, SN; Submandibular Neck, TP; Temporoparietal Neck, PM; Postauricular Mastoid, FA; Forearm. Scale bar = 50 μm. (ZIP 2133 kb) [file 40463_2017_210_MOESM4_ESM.zip › Slide10.tiff]
